# Complete plastome sequence of *Bridelia tomentosa* Blume (Phyllanthaceae): a medicinal shrub species in South Asia

**DOI:** 10.1080/23802359.2021.1951134

**Published:** 2021-07-15

**Authors:** Yan Chen, Xiu-Rong Ke, Xiao-Feng Zhang, Zhi-Xin Zhu, Hua-Feng Wang

**Affiliations:** Hainan Key Laboratory for Sustainable Utilization of Tropical Bioresources, College of Tropical Crops, Hainan University, Haikou, China

**Keywords:** *Bridelia tomentosa*, Euphorbia, Plastome, genome structure

## Abstract

*Bridelia tomentosa* is a deciduous shrub in the family of Phyllanthaceae. It grows in the evergreen primary or secondary thickets or forests in the sea level from 1000 to 1500 m. It distributed in.south China (e.g., Fujian, Guangdong, Guangxi, Hainan etc) and other south Asian countries (e.g. Bangladesh, Bhutan, Cambodia etc). Here, we report and characterize the complete plastome of *B. tomentosa*. The complete plastome is of 149,958 bp in length with a typical structure and gene content of angiosperm plastome, including two inverted repeat (IRs) regions of 26,354 bp, a large single-copy (LSC) region of 81,355 bp and a small single-copy (SSC) region of 15,895 bp. The plastome contains 129 genes, consisting of 84 protein-coding genes, 37 tRNA genes, eight rRNA genes. The overall G/C content in the plastome of *B. tomentosa* is 36.0%. The complete plastome sequence of *B. tomentosa* will provide a useful resource for the conservation genetics of this species as well as for phylogenetic studies in Phyllanthaceae.

## Introduction

*Bridelia tomentosa* Blume is a deciduous shrub in the family of Phyllanthaceae. It grows in the evergreen primary or secondary thickets or forests in the sea level from 1000 to 1500 m. It distributed in.south China (e.g. Fujian, Guangdong, Guangxi, Hainan etc) and other south Asian countries (e.g. Bangladesh, Bhutan, Cambodia etc). The leaves of *B. tomentosa* could be used as medicine for traumatic injury. Besides, its roots could be used to treat epidemic influenza and neurasthenia and its bark yields up to 8% tannin (Li et al. 2008). Therefore, we reported the complete plastome of *B. tomentosa* in this study, (GenBank accession number is MW357611), which expect to improve the quality of relevant collection, medical application and phylogenetic investigation of Phyllanthaceae.

In this study, *B. tomentosa* was sampled from Ruili county in Yunnan (97.84°E, 24.00°N). A voucher specimen (voucher code: RL0909, Wang et al.) and its DNA was deposited in the Herbarium of the Institute of Herbarium of China National GeneBank (code of herbarium: HCNGB).

The experiment was carried out as reported in Zhu et al. (2018). With MITO bim v1.8 (le-petit-quevilly, France) (Hahn et al. 2013), about six Gb of cleaning data was assembled for the plastid group of *Populus lasiocarpa* KX641589.1 (Rivarola et al. 2011). Using Geneious R8.0.2 (Biomatters Ltd., Auckland, New Zealand), the plastome was annotated against the plastome of *Populus lasiocarpa* (KX641589.1). The annotation was corrected with DOGMA (Wyman et al. 2004).

The result of our study indicates that the complete length of the plastome of *B. tomentosa* possesses 149,958 bp with the typical quadripartite structure of angiosperms, which includes two Inverted Repeats (IRs) of 26,354 bp, a Large Single-Copy (LSC) region of 81,355 bp and a Small Single-Copy (SSC) region of 15,895 bp. The plastome contains 129 genes, consisting of 84 protein-coding genes (seven of which are duplicated in the IR), 37 tRNA genes (seven of which are duplicated in the IR) and eight rRNA genes (5S rRNA, 4.5S rRNA, 23S rRNA and 16S rRNA) (four of which are duplicated in the IR). The overall G/C content in the plastome of *B. tomentosa* is 36.0%, which the corresponding value of the LSC, SSC and IR region were 33.7%, 30.1% and 41.4%, respectively.

**Figure 1.**
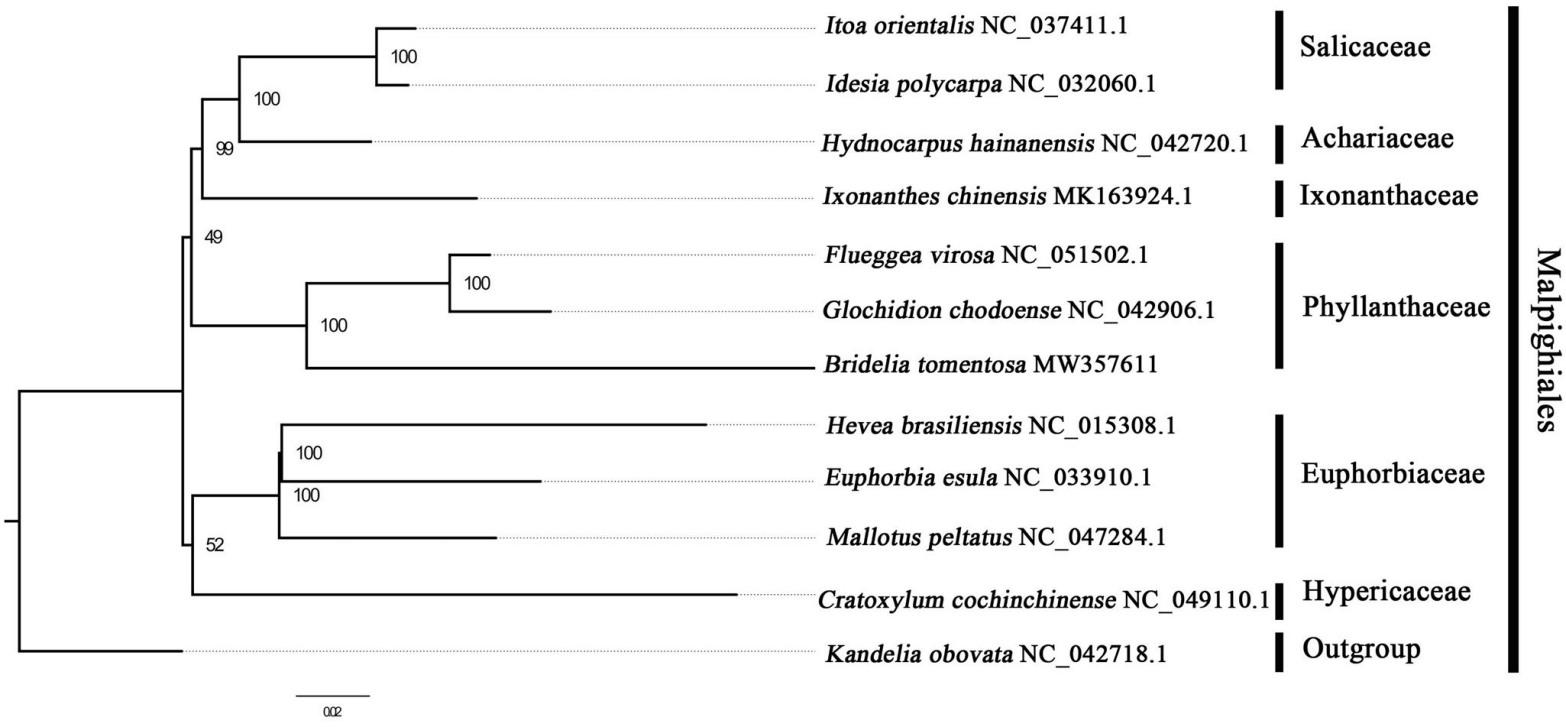
The best ML phylogeny recovered from 12 complete plastome sequences by RAxML. Accession numbers: *Bridelia tomentosa* (GenBank accession number, MW357611, this study), *Itoa orientalis* NC_037411.1, *Idesia polycarpa* NC_032060.1, *Hydnocarpus hainanensis* NC_042720.1, *Ixonanthes chinensis* MK163924.1, *Flueggea virosa* NC_051502.1, *Glochidion chodoense* NC_042906.1, *Hevea brasiliensis* NC_015308.1, *Euphorbia esula* NC_033910.1, *Mallotus peltatus* NC_047284.1, *Cratoxylum cochinchinense* NC_049110.1, *Kandelia obovata* NC_042718.1.

We used RAxML (Stamatakis 2006) with 1000 bootstraps under the GTRGAMMAI substitution model to reconstruct a maximum likelihood (ML) phylogeny of eleven published complete plastomes of Malpighiales, using *Kandelia obovata* NC_042718.1 as outgroups. By builting phylogenetic relationship based on the existing data and related taxa, we find that *B. tomentosa* is closer to *Flueggea virosa* and *Glochidion chodoense* than other species in this study (Figure 1). Current data and surveys can sufficiently show that the majority of nodes in the plastome ML trees were highly supported. Nowadays,the plastid sequence of *B. tomentosa* has been gradually developed and tended to be perfect,which does a great deal to promote the relevant conservation and phylogenetic investigation of Phyllanthaceae, it can bring great benefits to deepen the understanding of such plants of Phyllanthaceae.

## Data Availability

The genome sequence data supporting the results of this study are publicly trustworthy in GenBank of NCBI (https://www.ncbi.nlm.nih.gov/)with registration number MW357611. The associated BioProject, SRA, and Bio-Sample numbers are SRS3261052, PRJNA438407 and SAMN08770974 respectively.
